# Loss of *flfl* Triggers JNK-Dependent Cell Death in *Drosophila*

**DOI:** 10.1155/2015/623573

**Published:** 2015-10-25

**Authors:** Jiuhong Huang, Lei Xue

**Affiliations:** Institute of Intervention Vessel, Shanghai 10th People's Hospital, Shanghai Key Laboratory of Signaling and Diseases Research, School of Life Science and Technology, Tongji University, 1239 Siping Road, Shanghai 200092, China

## Abstract

*falafel* (*flfl*) encodes a *Drosophila* homolog of human SMEK whose *in vivo* functions remain elusive. In this study, we performed gain-of-function and loss-of-function analysis in *Drosophila* and identified flfl as a negative regulator of JNK pathway-mediated cell death. While ectopic expression of *flfl* suppresses TNF-triggered JNK-dependent cell death, loss of *flfl* promotes JNK activation and cell death in the developing eye and wing. These data report for the first time an essential physiological function of *flfl* in maintaining tissue homeostasis and organ development. As the JNK signaling pathway has been evolutionary conserved from fly to human, a similar role of PP4R3 in JNK-mediated physiological process is speculated.

## 1. Introduction

falafel (flfl) is a* Drosophila* protein phosphatase 4 (PP4) regulatory subunit 3 (PP4R3) [1], which specifically mediates Miranda (Mira) localization and determinants cell fate during both interphase and mitosis [2]. flfl binds to CENP-C with its EVH1 domain [3] that is crucial for PP4 catalytic activity to centromeres at chromosomes during mitosis. Previous study proposed that PP4 functions through the modular activity of its component subunits [3]. Although* in vitro* studies have reported that PP4 is involved in a variety of molecular and cellular processes including regulation of c-Jun N-terminal kinase (JNK) pathway [4], NF-kB pathway [5], hematopoietic progenitor kinase 1 [6], apoptosis [7], and cell division [8], flfl's* in vivo* functions remain poorly understood. The human homolog of flfl is SMEK, which recruits PP4c to promote neuronal differentiation by dephosphorylating Par3 [9]. However, other* in vivo* functions of SMEK remain largely elusive.

The JNK pathway is evolutionary conserved from* Drosophila* to mammal [10]. As its genome has low redundancy,* Drosophila* has been used as an excellent genetic model to study tumor necrosis factor- (TNF-) induced cell death in development. In* Drosophila*, the TNF ortholog Eiger (Egr) triggers cell death through its receptor Grindelwald (Grnd) [11], the E2 ubiquitin conjugating enzyme complex Bendless/dUev1a [12, 13], the E3 ubiquitin ligase dTRAF2 [14], the TAK1-associated binding protein 2 Tab2 [15], and the dTAK1-Hep-Bsk (*Drosophila* homologs of JNKKK-JNKK-JNK) kinase cascade [16, 17]. In developing eyes, ectopically expressing Egr by* GMR*-Gal4 (*GMR* > Egr hereafter) induces JNK-dependent cell death and produces small eyes in adult [16, 17].

To identify additional factors that regulate Egr-triggered JNK-mediated cell death, we performed a genetic screen for dominant modifiers of the* GMR* > Egr small eye phenotype. From the screen, we found that expression of flfl suppresses Egr-triggered cell death. On the other hand, knocking down* flfl* induced JNK activation and JNK pathway-dependent cell death, suggesting a physiological function of flfl in animal development. To our knowledge, this is the first report that flfl negatively regulate TNF-JNK signaling-induced cell death* in vivo*.

## 2. Materials and Methods

### 2.1. Drosophila Strains

All stocks were raised on standard* Drosophila* media, and crosses were performed at 25°C.* UAS-flfl*-IR (V103793) was obtained from Vienna Drosophila Research Center,* UAS-flfl*-IR (31690), *flfl* ^EY03585^,* UAS*-*GFP*-IR, and* ap*-Gal4 were obtained from Bloomington Stock Center, and* UAS*-*bsk*-IR (5680R-2) was from Fly Stocks of National Institute of Genetics (NIG).* puc* ^*E69*^ [18],* GMR*-Gal4,* en*-Gal4,* pnr*-Gal4,* UAS*-GFP [19, 20],* UAS*-Egr [16],* UAS*-Egr^w^ [6],* UAS*-Hep, and* UAS*-Bsk^DN^ [21] were previously described.

### 2.2. AO Staining

Eye discs from 3rd instar larvae were dissected in 1% PBS buffer. AO staining procedure was based on previous assay [22]. Florescent image of eye discs labeled with AO was collected with Olympus Microscope BX51. 10 discs of each genotype were collected for statistics analysis.

### 2.3. Light Image

3-day-old flies of each genotypes were collected and immediately frozen at −80°C. For the image, flies were mounted on 1% agarose plates. Light images of eye and thorax were documented with OLYMPUS stereo microscope SZX16.

### 2.4. X-Gal Staining

X-Gal staining was performed as previously described with minor modification [23, 24]. Wing imaginal discs from 3rd instar larvae were dissected in 1% PBS buffer and fixed with 1% glutaraldehyde for 15 minutes at room temperature and incubated with *β*-galactosidase at 37°C for 24 hours.

### 2.5. Data Analysis

Invasive breast carcinoma stroma versus normal data was obtained from Oncomine database (https://www.oncomine.org/).

## 3. Results and Discussion

### 3.1. flfl Suppresses Egr-Induced Cell Death in Eye Development

As previous study showed, ectopic expression of Egr under the control of* GMR*-Gal4 induced a small eye phenotype [17]. This phenotype is mostly suppressed by coexpressing a dominant negative allele of Bsk (Bsk^DN^) encoding the* Drosophila* JNK ortholog [21], which indicates Egr-induced cell death is mainly mediated by JNK signaling [25]. To identify additional components of the Egr-JNK pathway or factors interacting with the pathway, we performed a genetic screen for dominant modifiers of the* GMR* > Egr small eye phenotype and identified Nopo, Ben, Wnd, and Wg signaling as essential regulator of Egr-JNK pathway induced cell death [21, 26].

**Figure 1:**
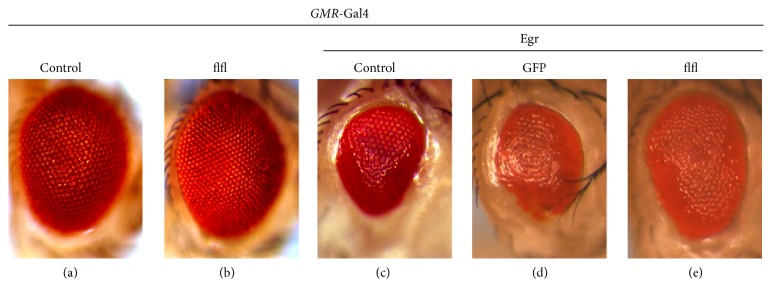
flfl suppress Egr-induced cell death in* Drosophila* eye. Light micrographs of* Drosophila* eyes are shown. Compared with the* GMR*-Gal4 control (a),* GMR* > Egr triggered cell death and produced a small eye phenotype (c), which was suppressed by expressing flfl (e) but not GFP (d). Expression of flfl produced no noticeable phenotype (b). Genotypes:* GMR*-Gal4/+ (a);* GMR*-Gal4/*flfl* ^*EY03585*^ (b);* UAS*-Egr/+;* GMR*-Gal4/+ (c);* UAS*-Egr/*UAS*-GFP;* GMR*-Gal4/+ (d);* UAS*-Egr/+;* GMR*-Gal4/*flfl* ^*EY03585*^ (e).

From the screen, we also found that the* GMR* > Egr small eye phenotype (Figure 1(c)) was significantly suppressed by *flfl* ^EY03585^ (Figure 1(e)), a P-element inserted in the first intron of* flfl*. This P-element carries the UAS sequence located about 1 kb upstream of the coding region and is able to drive the expression of flfl by the* GMR*-Gal4 driver. However, expression of* flfl* by itself had no effect on the eye size (Figure 1(b)), compared to the* GMR*-Gal4 control (Figure 1(a)). As a negative control, coexpressing GFP did not suppress* GMR* > Egr-triggered small eye phenotype (Figure 1(d)). Thus, the data indicate that flfl is able to suppress Egr-induced cell death in the eye.

### 3.2. Loss of* flfl* Enhances Egr-Induced Cell Death in Eye Development

**Figure 2:**
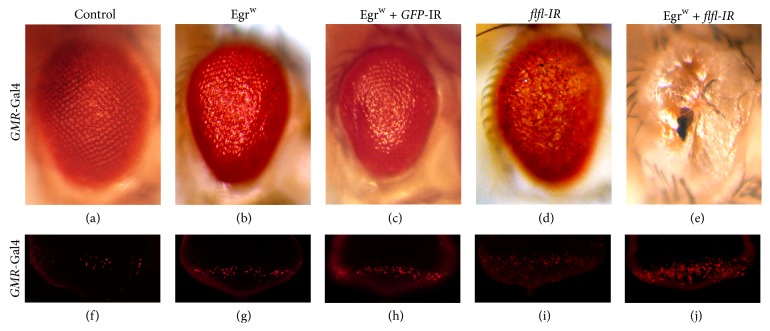
Loss of* flfl* enhances Egr-triggered cell death in the developing eye. Light micrographs of* Drosophila* eyes ((a)–(e)) or acridine orange staining of eye discs from 3rd instar larvae ((f)–(j)) are shown. Compared with the* GMR*-Gal4 control ((a) and (f)),* GMR* > Egr^w^ induced rough eye phenotype in adulthood (b) and cell death in larval eye disc (g) was not affected by expressing* GFP* RNAi ((c) and (h)) but was strongly enhanced by expressing* flfl* RNAi ((e) and (j)). Knocking down* flfl* alone caused a rough eye phenotype (d) and mild cell death in eye discs (i). Genotypes:* GMR*-Gal4/+ ((a) and (f));* UAS*-Egr^w^/+;* GMR*-Gal4/+ ((b) and (g));* UAS*-Egr^w^/*UAS*-*GFP*-IR;* GMR*-Gal4/+ ((c) and (h));* UAS*-*flfl*-*IR*/+;* GMR*-Gal4/+ ((d) and (i));* UAS*-Egr^w^/UAS-*flfl*-*IR*;* GMR*-Gal4/+ ((e) and (j)).

As flfl gain of function suppressed Egr-induced cell death, we wonder whether loss of* flfl* could enhance Egr-triggered cell death. To this end, we knocked down* flfl* in the eye by expressing* flfl* RNAi with* GMR*-Gal4 and observed a rough eye phenotype (Figure 2(d)), compared to the control (Figure 2(a)). Consistent with previous reports, expression of a weaker* UAS*-Egr allele (*UAS*-Egr^w^) driven by* GMR*-Gal4 resulted in a rough eye phenotype (Figure 2(b)). This phenotype is severely enhanced by knocking down* flfl* as there was almost no eye tissue left (Figure 2(e)). As a negative control, expressing a RNAi sequence specifically targeting green fluorescent protein (GFP) has no effect on GMR > Egr^w^-triggered rough eye phenotype (Figure 2(c)). These results show that flfl loss of function rigorously enhances Egr-triggered eye phenotype.

It was previously reported that ectopic Egr-induced eye phenotype is caused by cell death [16]. To examine cell death* in vivo*, we performed acridine orange (AO) staining that specifically labels dying cell. As reported previously [12], ectopic expression of a weak* UAS*-Egr transgene (*UAS*-Egr^w^) driven by* GMR*-Gal4 induced mild cell death in eye discs posterior to the morphogenetic furrow (MF), as revealed by AO staining (Figure 2(g)). Egr-triggered cell death was rigorously enhanced by expressing * flfl* RNAi (Figure 2(j)) but remained unaffected by expressing* GFP* RNAi (Figure 2(h)). Consistent with its rough eye phenotype, knocking down* flfl* provoked weak cell death (Figure 2(i)). These data suggest that loss of* flfl* enhances Egr-induced cell death in eye development.

### 3.3. Loss of* flfl* Enhances JNK-Mediated Cell Death in Thorax Development

**Figure 3:**
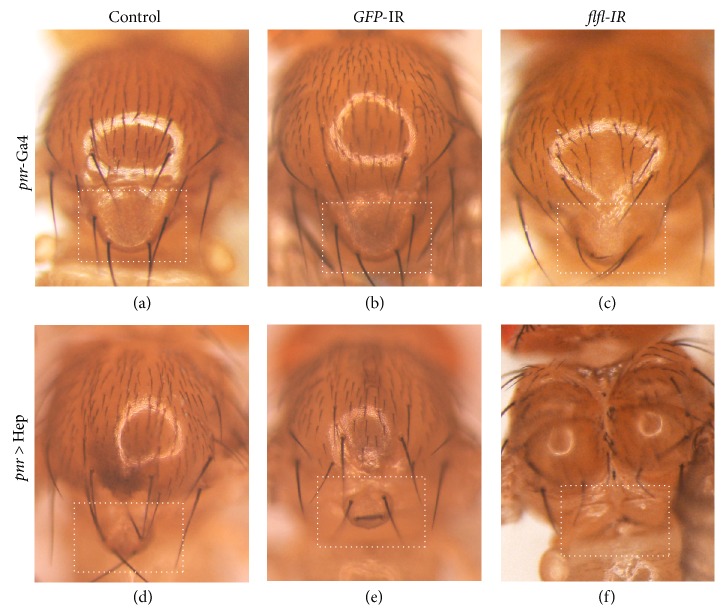
Loss of* flfl* enhances JNK-mediated cell death in thorax. Light images of* Drosophila* adult thoraxes are shown. Compared with the wild type (a) and* pnr* >* GFP*-IR control (b), expression of Hep induced a small scutellum (d), which was dramatically enhanced by the expression of* flfl* RNAi (f), while expression of* flfl* RNAi slightly decreased scutellum size (c). Dashed rectangle indicates the scutellum. Genotypes:* pnr*-Gal4/+ (a);* UAS*-*GFP*-IR/+;* pnr*-Gal4/+ (b);* UAS*-*flfl*-IR/+;* pnr*-Gal4/+ (c);* UAS*-Hep/+;* pnr*-Gal4/+ (d);* UAS*-Hep/*UAS*-*GFP*-IR;* pnr*-Gal4/+ (e);* UAS*-Hep/*UAS*-*flfl*-IR;* pnr*-Gal4/+ (f).

To investigate whether flfl suppresses JNK-mediated cell death in other tissues, we activated JNK signaling in the notum with* pannier*-Gal4 (*pnr*-Gal4). Expression of Hep, the* Drosophila* homolog of JNK, driven by* pnr*-Gal4 induced cell death and produced a small scutellum in adult fly (Figure 3(d)) [21]. Knocking down* flfl* by* pnr*-Gal4 slightly decreased scutellum size (Figure 3(c)) and dramatically enhanced Hep-induced cell death by producing a no scutellum phenotype as well as a split thorax in adult flies (Figure 3(f)). As a negative control, expression of a* GFP* RNAi did not produce any effect on scutellum size (Figures 3(b) and 3(e)). Together, the results indicated that flfl negatively regulates JNK-mediated cell death in thorax development.

During* Drosophila* imaginal discs development, slow-proliferating cells are eliminated by a process called “cell competition” [27], which regulates tissue's homeostasis and organs' fitness and final cell number. JNK pathway was shown to play a crucial role in cell competition by eliciting cell death in “loser cells” [28, 29]. Since our data suggest that flfl impedes JNK-mediated cell death in a nontissue specific manner, flfl is likely a negative regulator of JNK-dependent cell competition and tissue homeostasis.

### 3.4. Loss of* flfl* Induces JNK Pathway Activation and Cell Death in Wing Development

**Figure 4:**
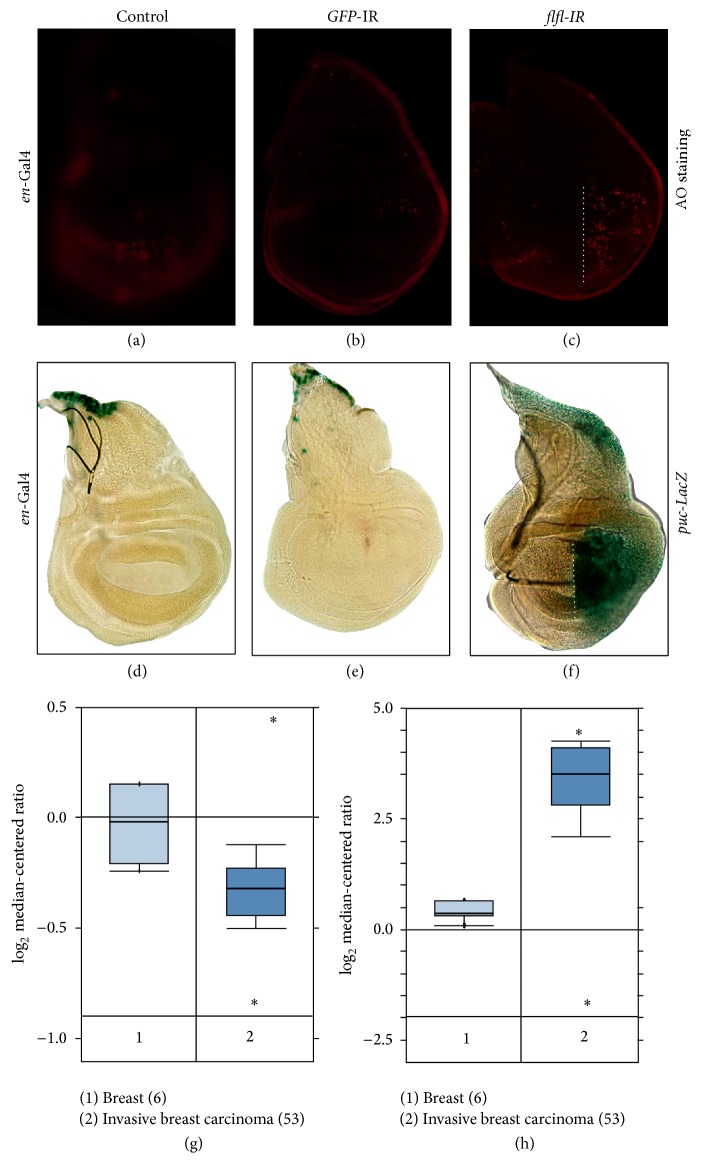
Loss of* flfl* induces JNK pathway activation and cell death in wing development.* Drosophila* 3rd instar wing discs with AO ((a)–(c)) and X-Gal staining ((d)–(f)) are shown. Knocking down* flfl* in the posterior compartment of wing discs by* en*-Gal4 induced extensively cell death (c) and* puc*-LacZ expression (f), while expressing a* GFP* RNAi failed to do so ((b) and (d)).* en*-Gal4 ((a) and (d)) served as controls. Dashed line indicates the anterior-posterior boundary of wing discs ((c) and (f)). Anterior boundary is to the left in all panels. Genotypes:* en*-Gal4/+ (a);* en*-Gal4/UAS-*GFP*-IR (b);* en*-Gal4/*UAS*-*flfl*-IR (c);* en*-Gal4/+; *puc* ^E69^/+ (d);* en*-Gal4/+; *puc* ^E69^/*UAS*-*GFP*-IR (e);* en*-Gal4/+; *puc* ^E69^/*UAS*-*flfl*-IR (f). SMEK1 (g) and DUSP1 (h) relative expression level in invasive breast carcinoma stroma compared to normal tissue in Finak Breast dataset are shown. Reporter: A_24_P36961 and A_23_P110712 are probes used in the study to detect SMEK1 and DUSP1, respectively. Breast stands for normal samples. The number in the parenthesis represents the total number of samples.

To investigate the physiological functions of* flfl* in wing development, we specifically knocked down* flfl* in the posterior compartment of wing discs by* engrailed*-Gal4 (*en*-Gal4) and checked cell death with AO staining. We found that loss of* flfl* triggered extensive cell death in the posterior compartment of wing discs (Figure 4(c)), compared with the* en*-Gal4 control (Figure 4(a)) and* en* >* GFP*-IR (Figure 4(b)). These results suggest that* flfl* is physiologically required for cell survival in* Drosophila* wing development.

To examine whether JNK signaling plays a role in loss of* flfl* induced cell death, we checked the expression of* puc*, a transcriptional target of JNK pathway [30].* puc* ^*E69*^ is a* puc* mutant allele with a LacZ bearing P-element inserted into the* puc* locus and serves as a* puc*-LacZ reporter [31] whose expression could be easily visualized by X-Gal staining. We found that knocking down* flfl* in the posterior compartment of wing discs resulted in upregulated* puc-LacZ* expression (Figure 4(f)), compared with the* en*-Gal4 control (Figure 4(d)) and* en* >* GFP*-IR (Figure 4(e)), suggesting that loss of* flfl* promotes JNK pathway activation.

The JNK pathway is evolutionary conserved from fly to human. Compared with the compact* Drosophila* genome, there are three homologs of flfl, SMEK1, SMEK2, and SMEK3P, and dozens of Puc homologs named dual specificity phosphatase (DUSP) in human. Previous study has reported that JNK signaling is essential for cell migration and tumor invasion [32]. Based on the above data, we speculate that SMEK is downregulated and DUSP is upregulated in metastatic tumor. Consistent with the hypothesis, we found from the Oncomine database (https://www.oncomine.org/) that SMEK1 expression is indeed downregulated whereas DUSP1 is upregulated in invasive breast carcinoma stroma compared to normal tissue (Figures 4(g) and 4(h)) [33]. These data imply that the role of flfl in modulating JNK pathway is likely conserved by SMEK1 from* Drosophila* to human.

Although our data mining and previous study found that JNK activity is elevated in several cancer cell lines, its role in tumor development is context-dependent [8]. JNK pathway was implicated as both procancer and anticancer signaling in cancer development for its regulation on cell proliferation and cell death, respectively [6]. In certain mouse models of cancer, JNK deficiency enhances tumor formation and metastasis [20, 34]. In* Drosophila*, clones with ectopic oncogene Src expression induce no-autonomous tumor growth [35], while Src expression also induces cell death through JNK pathway [22]. Cells in Src clone could escape from cell death if JNK pathway is blocked [35]. Intriguingly, another important oncogene Ras can also switch JNK pathway from anti- to protumor signaling [6]. Thus, upon the presence of different regulating factor(s), JNK pathway modulates cell death, tumor genesis, and progression in a cell context-dependent manner.

### 3.5. Loss of* flfl* Induced Cell Death Is JNK Pathway-Dependent

**Figure 5:**
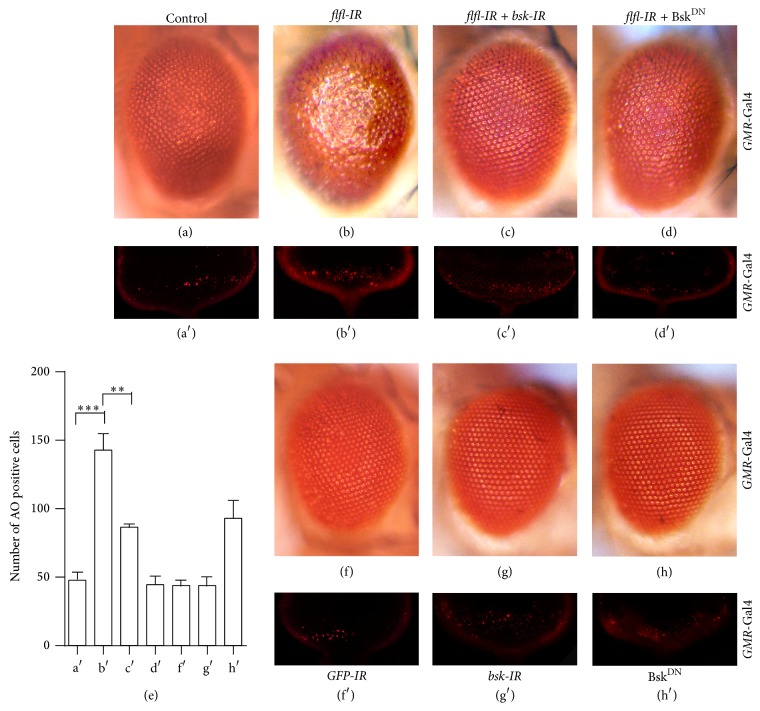
*flfl* loss-of-function induced cell death was suppressed by compromised JNK activity. Light micrographs of* Drosophila* eyes ((a)–(d) and (f)–(h)) or acridine orange staining of eye discs from 3rd instar larvae ((a′)–(d′) and (f′)–(h′)) are shown. Compared with the control (a), knocking down* flfl* induced cell death in eye discs (b′) and a rough eye phenotype in adult (b), which were significantly suppressed by knocking down* bsk* ((c′) and (c)) or coexpressing a dominant negative form of Bsk ((d′) and (d)). Expressions of* GFP*-IR,* bsk*-IR, or Bsk^DN^ were included as controls ((f)–(h′)). (e) is the statistical analysis of acridine orange positive cells in the posterior part of eye discs from the indicated panels. Column shows mean + SEM and significance was tested by unpaired Student *t*-test;^*∗∗∗*^ *P* ≤ 0.001; ^*∗∗*^ *P* ≤ 0.01.

Knocking down* flfl* by* GMR*-Gal4 induced cell death in eye discs (Figure 2(i)) and produced a rough eye phenotype in adults (Figure 2(d)). These results were confirmed by another independent line of* flfl* RNAi (Figures 5(b) and 5(b′)). To understand whether loss of* flfl *induced cell death is JNK pathway dependent, we blocked JNK signaling by expressing a bsk RNAi or a dominant negative allele of Bsk (Bsk^DN^). We found that loss of* flfl* triggered rough eye phenotype (Figure 5(b)) and increased cell death in eye discs (Figure 5(b′)) were significantly suppressed by compromised JNK activity (Figures 5(c)–5(e)). As a control,* GFP* RNAi and loss of Bsk signaling produced no evident phenotype in adult eyes (Figures 5(f)–5(h)). These results indicate that depletion of* flfl *induced cell death is JNK pathway-dependent.

## 4. Conclusions

In this study we have identified flfl as a negative regulator of TNF-trigger JNK-mediated cell death in* Drosophila*. While ectopic expression of* flfl* impedes JNK signaling-induced cell death, loss of* flfl* induces JNK pathway activation and cell death in* Drosophila* eye and wing discs and produced morphological defects in the adult eye. These data suggest an important physiological function of flfl in maintaining tissue homeostasis in* Drosophila* organ development. flfl's ability to inhibit JNK signaling is likely retained by its human homolog SMEK1. Consistently, while activated JNK pathway promotes dermal fibroblasts cell migration in wound healing [36], ectopic expression of SMEK1 significantly decreased the migration ability of carcinoma cells [37]. In addition, we found from Oncomine database that SMEK1 is downregulated whereas JNK signaling target gene DUSP1 is upregulated in human invasive carcinoma [33].
